# Inherited Retinal Diseases with High Myopia: A Review

**DOI:** 10.3390/genes16101183

**Published:** 2025-10-11

**Authors:** Cyndy Liu, Narin Sheri, Matthew D. Benson

**Affiliations:** Department of Ophthalmology and Visual Sciences, University of Alberta, Edmonton, AB T6G 2R3, Canada; cyndy@ualberta.ca (C.L.); narin@ualberta.ca (N.S.)

**Keywords:** inherited retinal dystrophy, retinal degeneration, high myopia, axial myopia

## Abstract

Inherited retinal dystrophies (IRDs) are a diverse group of monogenic disorders associated with dysfunction of the retina. High myopia, commonly defined as a spherical equivalent ≤ −6.00 D or axial length ≥ 26.5 mm, is a recurring clinical feature across several IRDs, and could serve as an early diagnostic clue. This review provides a summary of IRDs associated with high myopia to guide the clinician in establishing a molecular diagnosis for patients. We performed a comprehensive literature review of articles in PubMed, ScienceDirect, and JAMA Network to identify associations between monogenic IRDs and high myopia. Genes associated with IRDs and high myopia clustered into functional categories that included collagen/structural integrity (*COL2A1*, *COL9A1*, *COL11A1*, *COL18A1*, *P3H2*), phototransduction and visual cycle (*PDE6C*, *PDE6H*, *GUCY2D*, *ARR3*, *RBP3*), ciliary trafficking and microtubule-associated genes (*RPGR*, *RP2*, *IFT140*, *CFAP418*, *FAM161A*), synaptic ribbon and bipolar cell signaling (*NYX*, *CACNA1F*, *TRPM1*, *GRM6*, *LRIT3*, *GPR179*), opsin-related genes (*OPN1LW*, *OPN1MW*), and miscellaneous categories (*VPS13B*, *ADAMTS18*, *LAMA1*). Associations between IRDs and high myopia spanned stationary and progressive retinal disorders and included both cone-dominant and rod-dominant diseases. High myopia accompanied by other visual symptoms and signs such as nyctalopia, photophobia, or reduced best-corrected visual acuity should heighten suspicion for an underlying IRD. Earlier diagnosis of IRDs for patients could facilitate timely genetic counseling, participation in clinical trials, and interventions for patients to preserve vision.:

## 1. Introduction

Inherited retinal dystrophies (IRDs) are a group of disorders characterized by the progressive degeneration of the retina and/or retinal pigment epithelium (RPE) [1]. The retina contains cone and rod photoreceptors that are crucial for phototransduction, where light entering the eyes is converted into an electric signal for the brain to process [2]. The RPE is a single layer of cells that plays an important role in supporting the neural retina by forming the outer blood-retinal barrier, sustaining photoreceptors through the transportation of ions/nutrients, recycling vitamin A for the visual cycle, and managing waste products, such as reactive oxygen species (ROS) [3]. IRDs arise from pathogenic variants in genes expressed in the retina and/or RPE, impacting approximately 1 in 3000 individuals globally [4]. These conditions can cause progressive vision loss, and common symptoms can include: reduced central vision, light sensitivity, colour blindness, night blindness, loss of peripheral vision, and progression to complete blindness [5]. Currently, treatment options are limited, but a range of therapeutic approaches including small molecule therapies, genetic therapies, stem cell therapies, and retinal implants are being investigated in clinical trials [6]. An important aspect in managing patients with IRDs is the early detection of the condition, which may result in an improved prognosis through the identification and management of any associated ocular and systemic complications [7]. Early disease detection also helps identify at-risk family members for additional testing, can inform family planning through genetic counseling and helps position patients for participation in clinical trials. Therefore, it is important to explore different methods of improving the diagnosis and treatment of IRDs. There is a group of IRDs that are frequently associated with high myopia, which is typically defined as a refractive error of ≤−6.00 D or an axial length of ≥26.5 mm [8]. Identifying shared clinical signs, such as high myopia, can streamline diagnosis and inform potential shared underlying pathomechanisms, both of which may be important for developing treatments. In this review, we examine the correlation between IRDs and myopia by examining the genes associated with both high myopia and retinal dystrophies.

## 2. Methods

Although myopia is common globally, high myopia is estimated to affect 2–3% of the population [9]. Given the distinction between standard myopia and high myopia, there may be an association between high myopia and certain inherited retinal disorders. As a result, a comprehensive literature review was conducted using PubMed, ScienceDirect, and JAMA Network to identify genetic associations between inherited retinal dystrophies (IRDs) and high myopia. The search included keywords: high myopia, myopia, retinal dystrophies, retina, nearsightedness, and retinal degeneration. Ophthalmic measurements such as refractive error (≤−6.00 D) and axial length (≥26.5 mm) were considered when available. However, due to variability in definitions of high myopia, some case reports included patients who were just above the −6.00 D cut-off, and judgment was required in determining which genes to include in this review. We note that some patients may have other confounding reasons for their myopic refractive error, such as steep corneas. As well, some genes associated with myopia, but not high myopia, may not have been included. Additionally, there is a possibility that rare monogenic disorders that may be associated with high myopia are absent from this review due to limited availability of the literature on them. Despite these limitations, this review provides a guide for clinicians and vision scientists by focusing on overlapping molecular mechanisms to help refine differential diagnosis and potentially guide molecular testing for patients suspected of having IRDs.

**Figure 1:**
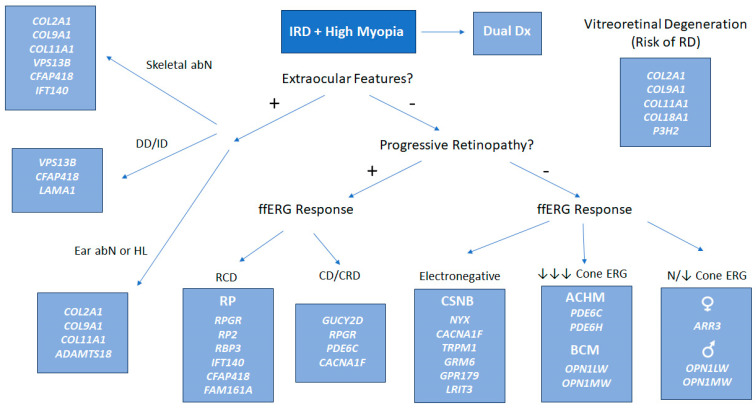
Diagnostic flowchart to assist in the clinical evaluation of patients presenting with an inherited retinal disorder (IRD) and high myopia. Abbreviations: ACHM = achromatopsia; BCM = blue cone monochromacy; CD = cone dystrophy; CRD = cone-rod dystrophy; CSNB = congenital stationary night blindness; DD = developmental delay; ERG = electroretinogram; HL = hearing loss; ID = intellectual disability; RCD = rod-cone dystrophy; RD = retinal detachment; RP = retinitis pigmentosa.

We have categorized our results according to the functional class of disorders that each gene belongs to (Table 1). In addition, we have included a flowchart based on clinical symptoms and signs which may assist clinicians in prioritizing molecular testing (Figure 1).

## 3. Collagen/Structural Integrity

### 3.1. COL2A1

The *COL2A1* gene encodes the alpha−1 chain of type II collagen, which is abundant in cartilage and vitreous humour of the eye [10]. Heterozygous pathogenic mutations in *COL2A1* are associated with type I Stickler syndrome which results from the improper formation of collagen [11]. Specifically, this genetic disorder can result in retinal abnormalities often presenting as optically empty or membranous vitreous (type I) with veils and perivascular and peripheral lattice degeneration [12,13]. Furthermore, sixty percent of patients develop rhegmatogenous retinal detachment (RD), the most common form of RD, where a tear or hole is present in the retina [14]. Pathogenic variants in *COL2A1* are also associated with other ocular conditions including glaucoma, cataracts, and lens subluxation [11,15]. Extraocular signs that would increase the suspicion for type 1 Stickler syndrome include midface hypoplasia, Pierre-Robin sequence (micrognathia, glossoptosis, cleft palate), hearing loss, and degenerative joint disease [16].

Kniest dysplasia is an allelic disorder that is associated with an ocular triad including high myopia, vitreous changes, and RD [17]. Spondyloepiphyseal dysplasia congenita (SEDC), also caused by heterozygous pathogenic *COL2A1* variants, demonstrates similar ocular features including high myopia, vitreous changes, and risk of RD [18].

Pathogenic variants in *COL2A1* are frequently associated with high myopia [19,20,21]. In addition, a large study of over 250 families with high myopia found a significant association with *COL2A1* single-nucleotide polymorphisms (SNPs) [22]. A recent study on 13 members of a four-generation Chinese family with early-onset high myopia utilized whole-exome sequencing (WES) to identify an intronic *COL2A1* variant accounting for the family’s high myopia [23].

### 3.2. COL9A1

*COL9A1* encodes one of the three alpha chains of type IX collagen [24]. Biallelic pathogenic mutations in *COL9A1* have also been associated with Stickler syndrome with ocular abnormalities, alongside more common causes of Stickler syndrome caused by mutations in *COL2A1* and *COL11A1* [25]. In *COL9A1*-related autosomal recessive Stickler syndrome, the vitreous does not phenocopy the classic type 1 Stickler syndrome (*COL2A1*; membranous vitreous) or type 2 Stickler syndrome (*COL11A1*; beaded vitreous) patterns. Rather, the vitreous is generally described as optically empty with a syneresis appearance due to progressive liquefaction [26].

One study evaluated a family including four children with *COL9A1*-related Stickler syndrome due to a novel homozygous nonsense variant, p.R295* [26]. Ophthalmic investigations of the four affected children revealed retinopathy in two children whose refractive errors were −15.00 D (right eye)/−14.50 D (left eye) and −4.50 D/−5.50 D. The other two affected children, who did not have retinopathies, had refractive errors of −4.00 D/−4.50 D and −3.50 D/−3.50 D [26].

Maghsoudi and colleagues reported on the risk of retinal detachment in a cohort of 13 patients (ages 3 to 42; median age 14) with *COL9A1*-related autosomal recessive Stickler syndrome [27]. All patients exhibited myopia (ranging from −2.00 D to −23.00 D), and 9 of the 13 patients had high myopia (−6.00 D or higher) in both eyes. Retinal detachment occurred in 2 out of 26 eyes (2 out of 13 patients; 15.5%) in the 3rd and 4th decade of life [27]. Given these findings, patients with *COL9A1*-related autosomal recessive Stickler syndrome may have a lower risk of retinal detachment compared to other types of Stickler syndrome. However, additional longitudinal studies with larger sample sizes are required to confirm these findings.

### 3.3. COL11A1

The *COL11A1* gene encodes for one of the two alpha chains in type XI collagen. Heterozygous pathogenic mutations in this gene are associated with type II Stickler syndrome, and patients characteristically have an irregular and beaded vitreous phenotype [28,29]. As in type I Stickler syndrome, the incidence of a retinal tear and detachment is increased [30]. Although retinal detachment in type II Stickler syndrome is less common than in type 1 Stickler syndrome, the risk remains approximately 40% and represents a significant cause of blindness in children [30].

One study evaluated four Turkish families with Marshall–Stickler syndrome and identified disease-causing *COL11A1* variants in three families, specifically 4 patients ranging from 1 month to 9 years of age [31]. Of the affected patients, 1 individual had degenerative myopia (−5.75 D/−5.25 D) and tigroid retinae, another unrelated patient also had myopia (refraction not reported), and a third unrelated patient exhibited severe myopia (−17.00 D/−17.00 D).

Given the substantial risk of RD in patients with Stickler syndrome, regular dilated peripheral retinal exams are prudent. Wide-field fundus imaging, if available, is helpful for documenting retinal pathology and evaluating for disease progression [32]. Importantly, an RD could present in infants or children who may be unable to verbalize any visual symptoms, so clinicians must maintain a high index of suspicion. There is a growing body of evidence to support prophylactic treatment, particularly peripheral laser retinopexy, in patients with Stickler syndrome [33,34,35,36]. A recent meta-analysis of 400 eyes of 225 patients with *COL2A1*- or *COL11A1*-related Stickler syndrome reported an RD rate of 6.6% (6.3 years mean follow-up duration) in patients who had prophylactic laser retinopexy compared to 36.0% (4.0 years mean follow-up duration) in patients without laser [36]. Importantly, the benefit of laser prophylaxis for mitigating RD was the greatest in patients with high myopia [36]. Consistent with previous studies, retinal detachment developed in 58.2% of patients with *COL2A1*-related Stickler syndrome who did not undergo laser prophylaxis, compared to 37.5% of patients with *COL11A1*-related Stickler syndrome. While patients with *COL2A1* pathogenic variants benefitted from laser prophylaxis, there were too few patients with *COL11A1* pathogenic variants (and too short of follow-up) to confirm whether a similar benefit exists. In summary, clinicians should consider offering prophylactic laser retinopexy for patients with Stickler syndrome, especially for patients with *COL2A1* pathogenic variants and high myopia [36].

### 3.4. COL18A1

*COL18A1* encodes the alpha chain of type XVIII collagen, which is important for brain development and the formation of many eye structures [37]. Specifically, type XVIII collagen is present in the corneal epithelial basement membrane and Descemet’s membrane, the basement membranes of the iris and ciliary body, and the retinal inner limiting membrane [38]. Biallelic pathogenic mutations in this gene cause Knobloch syndrome, a condition associated with vision abnormalities and occipital encephalocele [34]. Characteristic ocular features in Knobloch syndrome can include high myopia, vitreoretinal degeneration and malformations of the macula [39].

Similarly to *COL2A1*, biallelic pathogenic variants in *COL18A1* are consistently associated with high myopia as shown across cohort and case studies [40,41,42,43]. One case study examined 2 patients from a Chinese family where genetic testing had confirmed novel compound heterozygous *COL18A1* variants [42]. The 7-year-old patient had high myopia with a refractive error of −14.50 D in both eyes and an axial length of 22.3 mm (eye not specified). In addition, she had thinning of the retinae, but no retinal detachment at the time of the study. However, her 4-year-old sibling, who had a refractive error of −14.50 D/−15.50 D and an axial length of 21.36 mm (left eye only reported) had a history of total retinal detachment of the left eye and a partial retinal detachment of the right eye at the age of 7 months.

Recently, a novel variant in *PAK2* was identified in a newborn exhibiting features consistent with Knobloch syndrome, specifically atretic parietal meningocele and presumed retinopathy of prematurity (ROP) [44]. At 6–7 weeks of age, the patient was treated for rapidly progressive ROP, which was unresponsive to laser therapy. Subsequently, right vitrectomies were performed at 4 and 5 months of age for complex retinal detachments. Due to the ocular anomalies and meningocele, this study concluded that the kinase-deficient *PAK2* variant contributes to the pathogenesis of Knobloch syndrome, thus expanding the understanding of genes associated with syndromic retinal dystrophies.

### 3.5. P3H2/LEPREL1

*LEPREL1* encodes the enzyme prolyl 3-hydroxylase 2 (*P3H2*) that has an important role in the stabilization of connective tissue by catalyzing the post-translational inclusion of 3-hydroxyproline on collagens [45]. *P3H2* interacts with collagen COL4A1 and COL1A1 of the tendons, sclera, and membrane surrounding the lens of the eye [46]. Since *LEPREL1* plays a role in the formation of collagen, biallelic pathogenic loss-of-function mutations can result in ineffective collagen assembly that alters basement membrane structures of the eye, such as the inner limiting membrane (ILM). The ILM ensures proper development of the retina, and *LEPREL* mutations can weaken this layer, increasing the risk of retinal tears and subsequent retinal detachment [47]. *LEPREL1* mutations can also cause other ocular manifestations including high myopia.

A case study identified high myopia in a Bedouin Israeli family with homozygous *LEPREL1* mutations [48]. Ophthalmic exams were performed on 13 affected individuals, and refractive errors were reported in 11 patients, resulting in a mean refractive error of −11.30 D. All affected individuals demonstrated axial myopia, confirmed by elongated axial lengths ranging from 25.1 mm to 30.5 mm. Peripheral vitreo-retinal degenerative changes were present in 9 of the patients, of which 4 developed retinal tears resulting in RD in one or both of the eyes. Despite repeated surgical intervention, 3 of the patients had intractable RD which resulted in blindness.

## 4. Phototransduction and Visual Cycle

### 4.1. PDE6C

The gene *PDE6C* encodes the catalytic α’ subunit of the cone photoreceptor phosphodiesterase [49]. This enzyme is expressed in cone photoreceptors and facilitates colour vision and daylight vision. Biallelic pathogenic mutations in this gene typically cause achromatopsia, a condition characterized by complete or partial loss of colour vision, diminished visual acuity (VA), nystagmus, and photophobia.

There is evidence to suggest that pathogenic variants in *PDE6C* may be associated with high myopia. One study recruited 8 patients (ages 22 to 46 years old) with disease-causing mutations in *PDE6C* who all presented with early-onset fine nystagmus, decreased VA, light sensitivity, and severe color vision loss [50]. Ophthalmic investigations revealed that five of the eight participants had high myopia, with refractive errors of −10.25 D/−11.00 D, −10.50 D/−11.00 D, −14.00 D/−14.50 D, −11.00 D/−12.00 D, and −9.00 D/−9.75 D. Alongside the association with high myopia, 4 of the patients with *PDE6C*-associated achromatopsia were found to have outer retinal atrophy, which is a significant finding as *PDE6C* disease has been associated with progressive cone and cone-rod dystrophies [51]. While this study demonstrated an association between *PDE6C* and high myopia, achromatopsia (as a clinical diagnosis) is classically associated with hyperopia [52]. This association is likely driven by hyperopic refractive errors that tend to occur in patients with pathogenic variants in *CNGA3* and *CNGB3*, the most common genetic etiologies of achromatopsia [52].

### 4.2. PDE6H

*PDE6H* encodes the gamma subunit of the cone-specific cGMP phosphodiesterase. Biallelic pathogenic mutations in *PDE6H* are associated with achromatopsia, with ocular symptoms such as diminished visual acuity (VA) and photophobia (sensitivity to light) and abnormal colour vision [53].

Studies have also investigated the possibility of an association of *PDE6H* with high myopia. Andersen and colleagues conducted a study of Danish achromatopsia patients and compared refractive error across causal genes, including *PDE6H* [54]. Refractive error data were obtained from 82 individuals, 5 of whom had *PDE6H* variants. Of the 5 patients with *PDE6H* variants, 80% had high myopia. Furthermore, Kohl and colleagues reported refractive errors from 3 individuals with homozygous nonsense variants of *PDE6H*, which ranged from −6.50 D to 14.25 D [53]. Refractive errors in patients with achromatopsia appear to be gene-specific, where biallelic pathogenic variants in *PDE6C* and *PDE6H* tend to be associated with myopia.

### 4.3. GUCY2D

The gene *GUCY2D* encodes retinal guanylate cyclase-1, an enzyme that is expressed exclusively in the outer segments of photoreceptors and is crucial in the recovery phase of phototransduction [55]. The phototransduction cascade is initiated when light enters the eyes and stimulates specialized pigments known as opsins. When the opsins are exposed to light, a conformational change in the chromophore 11-cis-retinal to all-trans retinal occurs, triggering a cascade of events within the photoreceptor cells. The role of *GUCY2D* is to replenish cyclic guanosine monophosphate (cGMP), which is required to keep ion channels open in the photoreceptor cells. The cGMP produced by GUCY2D allows the photoreceptor cells to continue responding to light stimuli [56].

Pathogenic variants in *GUCY2D* can cause autosomal recessive Leber congenital amaurosis (LCA), as well as both autosomal dominant and recessive cone-rod dystrophy (CRD) [57]. LCA is a congenital disorder affecting the retina where patients frequently exhibit severely reduced visual acuity, night blindness, nystagmus, and an extinguished electroretinogram. There are over 25 genes associated with LCA [58]. CRD due to *GUCY2D* mutations is a type of hereditary retinal disorder that primarily affects the cone photoreceptors, with eventual involvement of the rod photoreceptors [59]. A clinical study featuring 42 patients with *GUCY2D* variants demonstrated that those with CRD had a higher prevalence of myopia (62% of participants) compared to other *GUCY2D*-related phenotypes [59].

While many types of LCA are characteristically associated with hyperopia, studies have suggested that *GUCY2D* could instead be associated with high myopia [60]. One study examined 50 patients with high myopia, and genetic testing determined that 4 of the patients had mutations linked with retinal dystrophies (*GUCY2D*, *FAM161A*, *PDE6H*, *CACNA1F*), suggesting an association between *GUCY2D* and high myopia [61].

### 4.4. ARR3

*ARR3* is expressed in cone photoreceptors and encodes for cone arrestin, which deactivates the phototransduction cascade after exposure to light [62]. Heterozygous pathogenic variants in *ARR3* are the most common cause of early-onset high myopia (eoHM) [63]. This condition typically presents in early childhood (before the age of 6) with symptoms of high myopia and reduced best-corrected visual acuity (BCVA) in a female-limited pattern [64]. In a study of 78 individuals from 29 families with *ARR3* mutations, 68 of the 78 patients were affected by eoHM, with refractive errors ranging from −5.00 D to −28.75 D [63]. Among the 78 patients, 64 out of 66 women and 4 out of 12 men were myopic, highlighting that *ARR3*-associated eoHM predominantly affects women. This predominant female X-linked inheritance is likely due to random X-inactivation in cones that causes cellular interference, whereas hemizygous males lack mosaicism and are often unaffected [64]. There are no overt differences in refraction, BCVA, or fundus features in affected females and males, however [63].

It is proposed that mutations in *ARR3* cause cone dysfunction and abnormal visual processing which leads to luminance contrast perturbations that drive the elongation of the eye. While *ARR3*-related eoHM appears to be distinct from cone dystrophies, patients frequently have reduced cone-driven responses on full-field electroretinography, leading us to include this gene in our review [63]. Given the X-linked inheritance pattern, we recommend that clinicians obtain a three-generation pedigree and document age of onset of myopia and sex distribution of affected relatives to guide molecular testing and genetic counseling.

### 4.5. RBP3

*RBP3* encodes for the interstitial retinol binding protein which is responsible for binding and transporting cis/trans retinols between the photoreceptors and the RPE [65]. *RBP3*-related retinopathy had been reported in three families, with one study reporting 4 siblings from an Italian family with autosomal recessive retinitis pigmentosa [66]. A second case reported on 3 children from 2 families who had generalized rod and cone dysfunction but had unremarkable fundus photos [67]. Furthermore, all eight affected patients in these studies had high myopia, thus suggesting an association between *RBP3*-related retinopathy and high myopia.

A recent study evaluated 12 patients with *RBP3*-related retinopathy and identified high myopia in all patients ranging from −7.00 D to −33.00 D [68]. The mean age of the cohort was 21.4 +/− 19.1 years (range from 2.9 to 60.5 years). All 12 patients had reduced visual acuity and an onset of myopia by the age of 7.

## 5. Ciliary Trafficking and Microtubule-Associated Genes

### 5.1. RPGR

The gene *RPGR* encodes a connecting cilium protein that supports intraflagellar transport in photoreceptors [69]. Loss-of-function of *RPGR* generally causes X-linked retinitis pigmentosa (XLRP); however, certain variants are more frequently associated with cone or cone-rod dystrophy [70]. While males are predominantly affected, females can present with a range of clinical signs that can occasionally be as severe as in males [71].

Patients with *RPGR*-related XLRP commonly exhibit high myopia, and studies have interrogated the association between *RPGR* mutations and high myopia [70,72,73]. One study recruited 17 patients (mean age at examination was 7.18 +/− 7.24 years) with X-linked retinopathies who underwent WES, Sanger sequencing, and ophthalmic investigations [74]. Of the 17 patients, 7 patients were confirmed to have *RPGR* mutations. It was determined that 57.1% of patients with *RPGR* mutations had high myopia (mean refractive error of the entire *RPGR* cohort was −5.29 D +/− 4.16 D; range from +1.50 D to −10.75 D), and 2 female patients with de novo *RPGR* variants had retinitis pigmentosa or cone-rod dystrophy along with high myopia. Axial length measurements were only available for 2 patients with *RPGR*-related retinopathy (24.74 mm/24.25 mm and 24.62 mm/24.55 mm) [74].

Another study evaluated a Chinese family with *RPGR*-related XLRP, where 8 patients were enrolled and underwent ophthalmic examination and genetic testing [75]. Three male patients (ages 32, 59, and 50 years) out of the eight were found to have high myopia with the following refractive errors: −10.00 D/−9.75 D, −9.25 D/−8.75 D, −5.75 D/−6.50 D, respectively. Additionally, one female carrier (55 years of age) had a refractive error of −1.25 D/−8.25 D. Other studies have also shown the association between *RPGR*-related XLRP and high myopia, with refractive error ranges reported as −10.75 D to +1.50 D in the *RPGR* subset of a Chinese X-linked retinopathies cohort [74], and −8.00 D to +1.00 D in affected males from a Japanese *RPGR* cohort (left eyes −7.00 D to +1.00 D) [76]. Within the Chinese cohort, 4 out of 7 patients had high myopia, while 50% of patients had high myopia within the Japanese cohort.

### 5.2. RP2

*RP2* encodes a GTPase-activating protein for ARL3 that supports ciliary protein trafficking in photoreceptors, and loss-of-function of *RP2* causes XLRP [77]. *RP2*-related retinopathy has been reported across many families and is characterized by early macular involvement and frequent high myopia [78]. Jayasundera and colleagues completed a cohort study of patients with *RP2* mutations and discovered 9 out of 11 affected males (ages all stated as less than 12 years old) were myopic with a mean refractive error of −7.97 D, and all 3 female carriers (ages 6, 28, and 45 years old) were myopic (mean −6.23 D) and had macular atrophy in one or both eyes [79]. Alongside the high myopia, electroretinography was conducted on 10 patients, which demonstrated severe rod-cone dysfunction in 9 out of 10 patients.

### 5.3. IFT140

*IFT140* encodes for one of the subunits of the intraflagellar transport system. Biallelic pathogenic variants in *IFT140* are associated with severe syndromic ciliopathies [80]. These disorders include Mainzer–Saldino syndrome and Jeune asphyxiating thoracic dystrophy, which can present with ophthalmic manifestations including retinitis pigmentosa and non-specific retinal dystrophies. A pediatric study investigated a 5-year-old patient diagnosed with Mainzer–Saldino syndrome who presented with retinitis pigmentosa [81]. Ophthalmic evaluation revealed an initial refractive error of −4.50 D/−6.25 D, and by 8 years of age his myopia progressed to −10.25 D/−11.00 D. Importantly, refractive errors generally progress as the eye grows; thus, genes associated with high myopia may only become evident as a child grows older.

### 5.4. CFAP418 (C8orf37)

*CFAP418* encodes for a protein that interacts with polyglutamylated tubulin at the base of the primary cilium in retinal pigment epithelium. Biallelic pathogenic mutations in *CFAP418* cause Bardet-Biedl syndrome and isolated retinopathies including cone-rod dystrophy (CRD) and retinitis pigmentosa (RP) [82]. One study investigated 2 Japanese siblings who presented with early-onset retinal dystrophy due to novel variants in *CFAP418* [83]. The siblings underwent ophthalmic exams which revealed decreased visual acuity, markedly reduced peripheral vision, high myopia, cataracts, and retinal degeneration with macular atrophy. Refractive errors were obtained at the age of 37 and 43 for the siblings and measured: −14.00 D/−9.00 D and −9.00 D/−10.00 D. Goyal and colleagues investigated two siblings with homozygous *C8orf37* mutations from a consanguineous North Indian family [84]. Both siblings had retinitis pigmentosa with early macular degeneration and night-vision loss in late childhood. Refractive errors obtained at the ages of 20 and 17, respectively, were −4.50 D/−5.50 D and −10.00 D/−10.00 D for each sibling [84].

### 5.5. FAM161A

*FAM161A* is a gene that is predominantly expressed in the retina and causes autosomal recessive retinitis pigmentosa [85]. *FAM161A* encodes a ciliary, microtubule-associated protein that plays a role in photoreceptor cells of the retina [86]. Various studies have shown an association between *FAM161A* mutations and high myopia [61,85,87].

*FAM161A* is the most common cause of autosomal recessive RP in Israeli-Jewish families, with one study examining refractive errors in 114 Israeli-Jewish patients with pathogenic biallelic mutations in *FAM161A* [87]. Of the 63 patients (ages ranging from 3.5 to 86 years old) for whom refractive data was obtained, all patients had varying degrees of myopia, ranging from −0.75 D to −15.00 D. Additionally, the majority of these patients had high myopia with the mean refraction of the cohort being −6.20 D ± 2.93 D.

Another study examined 50 patients with high myopia who all underwent WES [61]. Disease-causing mutations in *FAM161A* were identified in 1 patient; however, more gene-specific studies should be performed to highlight and validate the association of *FAM161A* with high myopia.

## 6. Synaptic Ribbon and Bipolar Cell Signalling

### 6.1. NYX

The *NYX* gene encodes for the protein nyctalopin, which is located at the synapse between photoreceptors and on-bipolar cells. Hemizygous pathogenic variants in *NYX* cause X-linked complete congenital stationary night blindness type 1 (CSNB1) [88]. CSNB1 is an inherited retinal disorder characterized by blindness in dimly lit conditions, myopia, nystagmus and reduced VA. High myopia is commonly found in patients with CSNB1. Poels and colleagues found that all (43 out of 43) patients older than 4 years with CSNB1 were myopic, with a mean refractive error of −7.50 D and a median refractive error of −8.10 D [89]. There was minimal progression of myopia after age 4 (approximately −0.12 D per year) [89,90]. Consistent with this, two Chinese families with CSNB1 with novel *NYX* variants had early-onset myopia that ranged from −4.5 D to −10.00 D, and one 11-year-old that had axial lengths 26.72 mm/26.65 mm [91]. Additionally, studies investigating patient cohorts with high myopia (and without known CSNB1) have identified variants in *NYX* [92].

For example, a study by Zhang and colleagues recruited 52 male probands who all had high myopia but did not have CSNB1 (or any other inherited retinal dystrophy) [92]. Two novel mutations in *NYX* were identified, suggesting that *NYX* mutations could cause isolated high myopia. It is suggested that disruptions in *NYX* cause aberrant retinal signalling, mimicking retinal blur that could cause abnormal elongation of the eye and subsequent high myopia.

### 6.2. CACNA1F

*CACNA1F* is a gene that encodes a voltage-gated calcium channel subunit in photoreceptors. Hemizygous pathogenic mutations in *CACNA1F* cause X-linked disorders including incomplete congenital stationary night blindness type 2 (CSNB2), cone-rod dystrophy (CORDX3) and Aland Island eye disease (AIED) [93,94]. *CACNA1F* has a well-established association with high myopia in affected males, and subsequent cohort studies confirmed high rates of myopia that often progress during childhood and throughout adolescence [89,94,95,96]. One study investigated 42 patients with *CACNA1F* mutations and reported 11 patients with a CSNB2 phenotype, 20 patients with a CORDX3 phenotype, and 11 patients with an AIED phenotype [94]. Importantly, all *CACNA1F*-related phenotypes are associated with high myopia. For the CSNB2 patients, the mean refractive error was −8.09 D ± 3.15 D. The CORDX3 and AIED patients had milder degrees of myopia, where the CORDX3 patients had a mean refractive error of −1.43 D ± 3.81 D, and the AIED patients had a mean refractive error of −1.82 D ± 4.70 D. This study suggested that specific mutations in *CACNA1F* in CSNB2 patients may uniquely contribute to the severity of myopia.

Another gene associated with incomplete CSNB (or cone-rod synaptic disorder) is *CABP4*, which encodes calcium-binding 4 and regulates Cav1.4-mediated calcium influx at photoreceptor ribbon synapses [97]. Unlike the association between *CACNA1F* and high myopia, the few published studies of *CABP4*-related disease report on patients with hyperopic refractive errors [97,98,99].

### 6.3. TRPM1

The *TRPM1* gene encodes a protein that plays an important role in signalling for bipolar cells in the retina, with biallelic pathogenic variants causing complete congenital stationary night blindness type 1C (CSNB1C) [100]. One study investigated 4 patients (two sibling pairs) from two families who underwent genetic testing and ophthalmic evaluations for presumed CSNB [101]. Genetic testing showed one sibling pair had a novel homozygous missense variant in *TRPM1*, while the other pair had a homozygous deletion. Three of the four patients had their refractive errors measured at the first visit: −11.25 D/−12.00 D, −8.50 D/−9.75 D, −8.50 D/−8.25 D. The fourth patient, who did not have a refractive error measurement reported, had an axial length of 27.39 mm/26.69 mm. This study suggests that pathogenic variants in *TRPM1* are associated with high myopia in CSNB patients, where altered signalling in ON-bipolar cells may promote axial elongation of the eye, resulting in high myopia.

Another longitudinal study followed 7 pediatric patients with *TRPM1*-associated CSNB, where 5 of the 7 patients had progressive myopia, strabismus and nystagmus [102]. Ophthalmic evaluation showed an average final refractive error of −8.75 D for the 7 patients, with a range from −4.00 D to −14.00 D, at a mean age of 12 years.

### 6.4. GRM6

The *GRM6* gene encodes a glutamate receptor, mGluR6, which resides in the membrane of retinal ON-bipolar cells [103]. In low light levels, rod photoreceptors release glutamate at their ribbon synapses, which then binds to mGluR6 to stimulate ON-bipolar cells. Biallelic pathogenic variants in *GRM6* interrupt glutamate signalling and cause the characteristic symptoms of complete CSNB type 1B such as night blindness [104].

Sergouniotis and colleagues reported on nine patients from seven families (ages ranging from 7 to 75 years old) and identified a median spherical equivalent refractive error of −5.38 D (range from +0.25 D to −17.00 D) [105]. Five out of the nine patients (55.6%) had high myopia. In addition, in a study of 96 unrelated Chinese patients with high myopia, 3 novel variants in *GRM6* with predicted functional consequences were identified [106]. The authors suggested that the identification of rare variants in *GRM6* in patients with high myopia may indicate a role for *GRM6* in myopia development [106].

### 6.5. LRIT3

*LRIT3* encodes a synaptic transmembrane protein required for ON-bipolar cell signalling in the retina, and biallelic pathogenic mutations cause complete CSNB type 1F [107]. Zeitz and colleagues investigated 90 individuals with CSNB and identified pathogenic *LRIT3* variants in two unrelated probands [108]. The first proband had a refractive error of −26.00 D/−27.00 D at age 45, with prior retinal tears treated at age 25. The second proband had a refractive error of −7.00 D/−8.00 D at age 9 [108], suggesting an association between *LRIT3* and high myopia.

Additionally, Dan and colleagues identified novel compound heterozygous variants in *LRIT3* in a patient with CSNB and high myopia (−8.50 D/−8.75 D at age 9) [109]. Despite the paucity of reported patients with *LRIT3*-related CSNB, the published cases find high myopia to be a consistent feature [108,109].

### 6.6. GPR179

*GPR179* encodes an orphan G protein-coupled receptor expressed in postsynaptic ON-bipolar cells. Biallelic pathogenic variants in *GPR179* cause complete CSNB type 1E [110]. Audo and colleagues reported on 5 probands with biallelic pathogenic variants in *GPR179* [111]. Two of these patients were described as having high myopia (ages not specified), but no refractive errors or axial lengths were mentioned. Refractive error was not commented on in the remaining three probands [111]. Klooster and colleagues reported on 2 patients with CSNB due to pathogenic *GPR179* variants [112]. The first patient had a refractive error of −9.00 D/−8.50 D at age 18, and the second patient had a refractive error of −8.00 D/−7.00 D at age 15 [112].

Considering the association of other types of complete CSNB with high myopia, in conjunction with the limited published cases of *GPR179*-related complete CSNB reporting myopic refractive errors, there likely exists an association between *GPR179* and high myopia, but larger studies are needed to confirm this.

## 7. Opsin-Related Genes

### 7.1. OPN1LW

*OPN1LW* encodes the red cone photopigment/long wavelength-sensitive opsin (L-opsin) that is crucial for facilitating colour vision [113]. Mutations in this gene cause cone-based disorders such as protanopia, the inability to perceive red light, or protanomaly, a reduced sensitivity to red light. Hemizygous variants of *OPN1LW* are also a well-established cause of blue cone monochromacy (BCM) [114].

Some *OPN1LW* mutations are associated with high myopia, purported to be due to abnormal mRNA slicing, causing photoreceptors to lack photopigment [115]. Specifically, Neitz and colleagues analyzed 413 healthy male patients and determined that patients with significant amounts of *OPN1LW* exon 3 skipping were associated with high myopia [115]. Photoreceptors with exon 3 skipping exhibit lower levels of photopigment, while nearby unaffected cones have normal levels. These varying levels of photopigment amongst adjacent cone photoreceptors can cause abnormal contrast signals. It is thus hypothesized that the abnormal contrast signals due to exon 3 skipping results in axial elongation and the development of myopia.

Another study recruited 1226 families with early onset high myopia (eoHM) and performed WES to elucidate the genetic basis of eoHM [116]. The authors identified novel *OPN1LW* variants in 68 out of the 1226 families. The range of refractive errors of these 68 families was between −3.75 D and −22.00 D, with a median refractive error of −9.38 D.

### 7.2. OPN1MW

*OPN1MW* is a gene encoding the green cone photopigment or medium-wavelength-sensitive opsin (M-opsin) [117]. On the X chromosome, one or more copies of *OPN1MW* are arranged in a head-to-tail opsin array immediately downstream of *OPN1LW.* The opsin *OPN1MW* is crucial for normal colour vision, specifically for facilitating sensitivity to yellow and green light. Mutations in the *OPN1MW* gene can cause abnormalities in colour vision, resulting in deuteranopia, an inability to perceive green light or deuteranomaly, a decreased sensitivity to green light. Additionally, hemizygous variants in *OPN1MW* are associated with BCM, which is strongly associated with high myopia in males [118,119].

In a multigenerational family with males who were hemizygous for the MVAVA haplotype of the *OPN1MW* gene, refractive error ranged from −5.00 D to −21.00 D [120]. In addition, the LVAVA haplotype of *OPN1LW* gene and MVAVA haplotype of *OPN1MW* have been reported to cause non-syndromic high myopia due to axial elongation in young patients [120]. Specifically, the MVAVA haplotype causes abnormal mRNA splicing and skips exon 3, which leads to loss of opsin function and cone dysfunction. Notably, specific exon 3 haplotypes in the *OPN1LW/OPN1MW* cluster (LIAVA, LVAVA and MVAVA) are known to cause defects in colour vision [121].

The L/M opsin array is prone to copy number variation (CNVs) and hybrid *OPN1LW* and *OPN1MW* genes from misalignment and unequal recombination due to their proximity and high sequence homology [122]. Routine short-read sequencing cannot readily differentiate between *OPN1LW* and *OPN1MW* and cannot readily detect structural variants and CNVs in the opsin array. As a result, targeted sequencing strategies such as MLPA and long-read sequencing are utilized for the molecular interrogation of the opsin array [122].

## 8. Miscellaneous

### 8.1. COH1/VPS13B

*VPS13B* (*COH1*) encodes a protein that plays an important role in intracellular transport [123]. Biallelic pathogenic variants in *VPS13B* cause Cohen syndrome, which is characterized by severe myopia and progressive retinal dystrophy. Extraocular manifestations of Cohen syndrome include: developmental delay, microcephaly, intellectual disability and truncal obesity with slender extremities [124,125]. One study conducted genetic testing and ophthalmic exams on 5 patients (4 females and 1 male) between the ages of 9 and 38 with Cohen syndrome [126]. Ophthalmic examinations revealed the following spherical corrections of the five patients: −10.00 D/−10.00 D, −10.00 D/−10.00 D, −14.50 D/−14.50 D, −3.25 D/−3.25 D, −6.25 D/−6.25 D, suggesting a correlation between *VPS13B* and high myopia. High myopia and chorioretinal dystrophy are considered the primary contributors to vision loss in Cohen syndrome, with another study reporting that patients typically exhibit a median refractive error of −8.00 D [127].

The dysregulation of protein trafficking arising from *VPS13B* variants is hypothesized to contribute to axial elongation and the development of high myopia in patients with Cohen syndrome [127].

### 8.2. ADAMTS18

*ADAMTS18* encodes a member of the ADAMTS protein family, with mutations being associated with an autosomal recessive disorder characterized by microcornea, myopic chorioretinal atrophy, and telecanthus (MMCAT) [128]. An investigation of 9 patients of Saudi ethnicity with molecularly confirmed MMCAT reported refractive errors in 7 patients (ages 4 to 10 years; mean spherical equivalent refractive error of −6.66 D; range from −0.50 D to −13.75 D) [128,129]. Four out of 7 (57.1%) patients had high myopia. Axial length measurements were reported for 6 patients, with a mean axial length of 24.63 mm (range from 22.41 mm to 26.84 mm) [128,129]. These findings suggest an association between variants in *ADAMTS18* and high myopia. Additional studies in diverse patient populations may further establish this association.

### 8.3. LAMA1

*LAMA1* encodes one of the alpha 1 subunits of laminin, and biallelic pathogenic variants in this gene cause Poretti–Boltshauser syndrome, a disorder associated with high myopia and variable retinal dystrophy [130].

One case study examined a 3-year-old boy with pathogenic variants in *LAMA1*, who underwent comprehensive ophthalmic testing [131]. Imaging reviewed retinal vascular anomalies such as a large area of nonperfusion in the temporal macula with corresponding retinal thinning. Furthermore, he had a refractive error of −16.50 D in each eye, highlighting the presentation of high myopia alongside retinal vascular abnormalities. Finally, another study identified five single-nucleotide polymorphisms (SNPs) in the *LAMA1* gene among 97 Chinese individuals diagnosed with high myopia, providing additional evidence to support an association between variants in *LAMA1* and high myopia [132].

## 9. Limitations

Our review highlights IRDs that are associated with high myopia, but it does not serve as an exhaustive list of all genes associated with high myopia. Not all studies of IRDs report refractive errors, and thus, it is possible that other IRDs may be associated with high myopia but are not included in our study. In addition, nystagmus is a feature of several of the IRDs included in our review, and this may reduce the accuracy of refraction measurements. Some case studies that we evaluated included patients who did not meet the threshold refractive error of ≤−6.00 D. These cases were included because there was a well-established association between the gene and high myopia and/or a substantial number of patients had high myopia, even if some individuals in the studies were just below this threshold. Furthermore, the lack of consistent data on axial length and keratometry in many studies introduces a gap in understanding the structural contributions to myopia development. Additionally, within families, it is unclear if high myopia is directly related to the underlying retinal dystrophy or due to other genetic or environmental factors. A degree of variability in refractive errors in patients with the same genetic variant is expected as there are multiple environmental and genetic factors that influence ocular growth and myopia development [133]. In addition, myopia typically progresses with age during childhood and adolescence. As a result, studies that reported on infants with retinal dystrophy and myopia > −6.00 D may not have been included in our study, but it is possible that the myopia in these patients may progress and reach the threshold of high myopia in the future. Moreover, ocular procedures such as cataract surgery and refractive surgery may make it challenging to identify genetic associations with high myopia if axial lengths and keratometry are not reported. Finally, there is a possibility that rare monogenic disorders are under-represented or absent in our study due to limited availability of published reports. Despite these limitations, our review can aid clinicians by guiding molecular testing for patients with IRDs in the setting of high myopia. In addition, by examining the overlap between molecularly confirmed IRDs and high myopia, this review also has the potential to enhance our understanding of the underlying mechanisms for eye growth and myopia development.

## 10. Conclusions

Our review highlights the molecularly confirmed IRDs that are associated with high myopia, emphasizing how shared genetic mechanisms may contribute to both retinal disease progression and the development of myopia. High myopia in the setting of other visual symptoms such as nyctalopia could serve as a clinical indicator to facilitate the early detection of IRDs, thus aiding clinicians in generating a differential diagnosis and providing effective patient counseling. Establishing an early diagnosis for patients with IRDs could allow for timely interventions, as there are numerous ongoing clinical trials and therapies on the horizon. Finally, interrogating the molecular mechanisms driving the development of high myopia is critical as myopia is a vision-threatening public health concern on a global scale [134]. Utilizing IRDs associated with high myopia as a lens to understand the genetic factors contributing to myopia may inform the development of novel therapies to combat the growing myopia epidemic.

## Figures and Tables

**Table 1:** Functional categories for IRDs associated with high myopia and monogenic high myopia.

Monogenic IRDs and High Myopia Functional Categories
Collagen/Structural Integrity
*COL2A1* (AD; OMIM: 120140)
*COL9A1* (AR; OMIM: 120210)
*COL11A1* (AD; OMIM: 120280)
*COL18A1* (AR; OMIM: 120328)
*P3H2* (AR; OMIM: 610341)
Phototransduction and Visual Cycle
*PDE6C* (AR; OMIM: 600827)
*PDE6H* (AR; OMIM: 601190)
*GUCY2D* (AD/AR; OMIM: 600179)
*ARR3* (XL; OMIM: 301770)
*RBP3* (AR; OMIM: 180290)
Ciliary Trafficking and Microtubule-Associated Proteins
*RPGR* (XL; OMIM: 312610)
*RP2* (XL; OMIM: 300757)
*IFT140* (AR; OMIM: 614620)
*CFAP418* (AR; OMIM: 614477)
*FAM161A* (AR; OMIM: 613596)
Synaptic Ribbon and Bipolar Cell Signalling
*NYX* (XL; OMIM: 300278)
*CACNA1F* (XL; OMIM: 300110)
*TRPM1* (AR; OMIM: 603576)
*GRM6* (AR; OMIM: 604096)
*LRIT3* (AR; OMIM: 615004)
*GPR179* (AR; OMIM: 614515)
Opsin-Related Proteins
*OPN1LW* (XL; OMIM: 300822)
*OPN1MW* (XL; OMIM: 300821)
Miscellaneous
*VPS13B* (AR; OMIM: 607817)
*ADAMTS18* (AR; OMIM: 607512)
*LAMA1* (AR; OMIM: 150320)

AD = autosomal dominant; AR = autosomal recessive; XL = X-linked; OMIM: Online Mendelian Inheritance in Man.

## Data Availability

No new data were created or analyzed in this study.
